# Specific, sensitive and rapid detection of human *plasmodium knowlesi *infection by loop-mediated isothermal amplification (LAMP) in blood samples

**DOI:** 10.1186/1475-2875-10-197

**Published:** 2011-07-20

**Authors:** Yee-Ling Lau, Mun-Yik Fong, Rohela Mahmud, Phooi-Yee Chang, Vanitha Palaeya, Fei-Wen Cheong, Lit-Chein Chin, Claudia N Anthony, Abdulsalam M Al-Mekhlafi, Yeng Chen

**Affiliations:** 1 Department of Parasitology, Faculty of Medicine, University of Malaya, 50603 Kuala Lumpur, Malaysia; 2 Institute for Research in Molecular Medicine (INFORMM), Universiti Sains Malaysia, 11800 Penang, Malaysia

## Abstract

**Background:**

The emergence of *Plasmodium knowlesi *in humans, which is in many cases misdiagnosed by microscopy as *Plasmodium malariae *due to the morphological similarity has contributed to the needs of detection and differentiation of malaria parasites. At present, nested PCR targeted on *Plasmodium *ssrRNA genes has been described as the most sensitive and specific method for Plasmodium detection. However, this method is costly and requires trained personnel for its implementation. Loop-mediated isothermal amplification (LAMP), a novel nucleic acid amplification method was developed for the clinical detection of *P. knowlesi*. The sensitivity and specificity of LAMP was evaluated in comparison to the results obtained via microscopic examination and nested PCR.

**Methods:**

LAMP assay was developed based on *P. knowlesi *genetic material targeting the apical membrane antigen-1 (AMA-1) gene. The method uses six primers that recognize eight regions of the target DNA and it amplifies DNA within an hour under isothermal conditions (65°C) in a water-bath.

**Results:**

LAMP is highly sensitive with the detection limit as low as ten copies for AMA-1. LAMP detected malaria parasites in all confirm cases (n = 13) of *P. knowlesi *infection (sensitivity, 100%) and none of the negative samples (specificity, 100%) within an hour. LAMP demonstrated higher sensitivity compared to nested PCR by successfully detecting a sample with very low parasitaemia (< 0.01%).

**Conclusion:**

With continuous efforts in the optimization of this assay, LAMP may provide a simple and reliable test for detecting *P. knowlesi *malaria parasites in areas where malaria is prevalent.

## Background

Malaria still poses a major threat in most parts of the world. It is widespread in tropical and subtropical regions, including parts of America, Asia and Africa. *Plasmodium knowlesi *is one of the simian malaria that causes human infection. In 2004, a large focus of human *P. knowlesi *infection was reported in the Kapit Division of Sarawak. It was reported that 101 out of 141 (71.6%) of human malaria cases at Kapit Hospital, which had been identified by microscopy as single *Plasmodium malariae *infections, were actually *P. knowlesi *and other non-*P. malariae *species by nested polymerase chain reaction (PCR) assays [1]. More recently, out of 111 human blood samples received (July 2006 - March 2008) by the Institute for Medical Research (IMR), 62 (55.9%) were positive for *P. knowlesi *by nested PCR. Positive *P. knowlesi *cases were observed in most states in Peninsular Malaysia [2].

Microscopic examination has been the mainstay of malaria diagnosis. Microscopy is relatively inexpensive, rapid, and relatively sensitive procedure when used appropriately. However, interpretations of smears require considerable expertise particularly at low-level parasitaemia. This could potentially lead to false negative results or unreliable species determination. The emergence of *P. knowlesi *in humans, which is in many cases misdiagnosed by microscopy as *P. malariae *due to the similarity in morphology has contributed to the needs of detection and differentiation of malaria parasites [3].

To date, nested PCR targeted on Plasmodium ssrRNA genes has been described as the most sensitive and specific method for Plasmodium detection. Genus- and species-specific primers have been used to amplify ssrRNA genes to detect mixed infections. However, this method is costly and requires trained personnel for its implementation.

Serology methods to detect antibodies by immunofluorescence or ELISA have been used for seroepidemiological studies of malaria. However, the difficulty of blood stage cultivation of the parasite has been hindering the use of these techniques.

Loop-mediated isothermal amplification (LAMP) which was originally developed by Notomi *et al *[4], is a very sensitive, easy and time saving method. The LAMP method can amplify up to 10^9 ^copies of targeted gene in less than an hour under isothermal conditions (65°C). A simple incubator, such as water bath or heating block is sufficient for the DNA amplification, which makes its use under field conditions feasible. The method uses four to six primers that recognize six to eight regions of the target DNA eliminating non-specific binding and thus ensuring the specificity of LAMP.

Of late, this method has been proven to be a powerful diagnostic tool in the detection of many parasitic infections in both human and animal models. In this study, a LAMP assay was developed for the detection of *P. knowlesi *genetic material targeting the apical membrane antigen-1 (AMA-1) gene. AMA-1 found in all *Plasmodium *parasites during the late schizont stage. This protein was reported to play a prominent role in the invasion of the host cell [5].

## Methods

### Clinical samples

Fifty-four malaria positive blood samples were collected from University Malaya Medical Centre (UMMC), Malaysia. Samples were further diagnosed by microscopy and nested PCR assay. Twenty blood samples were also collected from healthy donor. Ethical approval for this study was obtained from the Medical Ethics Committee of University Malaya Medical Centre.

### Microscopy

Thick and thin blood films were prepared and examined by skilled personnel with extensive experience in the identification of malaria parasites. Parasitaemia was assessed either per 1,000 erythrocytes in the thin film at low parasitaemia or per 200 white blood cells (WBC) in the thick film. In case of a putative negative film, it was considered negative if no parasites were seen after 500 leukocytes were counted.

### Rapid diagnostic kit

BinaxNOW Malaria Kit (Inverness Medical International, United Kingdom) was performed according to manufacturer's instructions and adapted using a drop of EDTA anticoagulated whole blood from microscopy confirmed cases. Test results were analysed after 15 minutes as recommended by the manufacturer's manual.

### DNA Extraction

Template DNA for LAMP and nested PCR assay were prepared using DNeasy Blood & Tissue Kit (QIAGEN, Valencia, CA). EDTA anticoagulated whole blood (200 μl) was extracted according to the provided protocol to give 100 μl of purified template DNA. Purified DNA was stored at -20°C.

### Nested PCR assay

The species of the malaria samples were determined by nested PCR assay. This assay targets the *Plasmodium *small subunit ribosomal RNA (*ssrRNA*) gene. The primers used for the nested PCR assay were identical to those previously published [1]. The reaction mixture for the first PCR step had 2 μl of DNA template, 250 nmol/L of each primer (*rPLU 1 *and *rPLU 5*, PCR buffer (50 mmol/L KCl, 10 mmol/L *Tris*-HCl), 200 mmol/L of each deoxynucleoside triphosphate, 1.25 units of *Taq *DNA polymerase (iDNA, Singapore), and water to a final volume of 25 μl. Primary amplification conditions were 94°C for 4 minutes; 35 cycles at 94°C for 30 seconds, annealing at 55°C for 1 minute, extension at 72°C for 1 minutes; and final extension at 72°C for 4 minutes. Two microliters of the first amplification product was used as DNA template for each of the 20 ml secondary amplifications. The conditions and concentrations of the secondary amplification were identical to those of the primary except for the annealing temperature of 58°C and the amount of *Taq *Polymerase being 0.5 units. The PCR products of the secondary amplification were analysed by gel electrophoresis and stained with ethidium bromide.

### LAMP assay

The FIP, BIP, F3, and B3 primers were designed using the Primer-Explorer V3 software [6] based on the apical membrane antigen-1 (AMA-1) gene sequence of *P. knowlesi *(GenBank accession number XM_002259303) (Table 1). Loop primers (Loop-F and Loop-B) were designed manually. The 25 μL reaction mixture consisted of 1.6 μM FIP and BIP, 0.8 μM Loop-F and Loop-B, 0.2 μM F3 and B3, 20 mM Tris-HCl, 10 mM KCl, 8 mM MgSO4, 10 mM NH4SO4, 0.1% Tween 20, 0.8 M betaine, 1.4 mM deoxynucleotide triphosphates, 1 μL *Bst *DNA polymerase and 2 μL template DNA. The reaction mixture was incubated in a water bath at 65°C for 45-60 minutes. To prevent cross-contamination, different sets of pipettes and different work areas were designated for DNA template preparation, PCR mixture preparation and DNA amplification.

**Table 1:** Primers used in this study

Primer	Sequence (5'→3')
FIP	TTACAAACGTAAAAGTTGCAGGTACGATAAGGAGAGTATCAAATGTCCA
BIP	CTGTGTAGAGAAGAGAGCAGAAATTCCGGATTTTCATAATCCTCC
F3	GCCAAGGATATTTATCTCCAC
B3	CTTCTTCTTATGTTTGCCGT
Loop-F	GGAAATGTGTTCAGGCTCAC
Loop-B	CATAAAGGAAGAATTTAA

### Endpoint assessment

Turbidity of the reaction mixture was observed with the naked eye. Turbidity is based on the precipitation of magnesium pyrophosphate as a by-product of the reaction. Besides the assessment of turbidity by the naked eye, amplicons of the LAMP were also detected by adding 1.0 μL of 1/10-diluted original SYBR^® ^Safe DNA gel stain (Invitrogen, Carlsbad, CA) to the tubes and observed under UV. Solutions with amplicons showed stronger luminescence under UV compared to the solutions without amplicons. For further confirmation, some of the amplified LAMP products were analysed by gel electrophoresis. All reactions were analysed by 2.0% (wt/vol) agarose gel by electrophoresis in Tris-Acetate-EDTA (TAE) buffer stained with SYBR^® ^Safe DNA gel stain and positive results were identified by the appearance of typical ladder bands of various sizes.

### Positive control plasmid DNA

For sensitivity assessment, plasmids containing the target region of the AMA gene were constructed for *P. knowlesi *for use in the LAMP reaction. The target DNA sequence was amplified with two LAMP primers (primers F3 and B3) by PCR and was then cloned into the pGEMT cloning vector (Invitrogen, CA). Plasmid DNA purification was performed with a QIAprep Miniprep kit (QIAGEN, Hilden, Germany). The resulting sequences were aligned using the AMA sequences for *P. knowlesi *in GenBank to confirm that the target sequences were correct.

### Analytical sensitivity of LAMP primers

Positive control plasmid DNAs were used to determine the minimum copy number (lower detection limit) of the target gene sequence detectable by LAMP. The standard curve for LAMP was constructed using 10-fold serial dilutions of plasmid DNA (10^6 ^copies to 1 copy) to sterile water. The copy number was plotted against the threshold time. The resulting plot was analysed by linear regression and the statistical significance of the *r*2 values was analysed by analysis of variance (ANOVA). Probabilities of less than 0.05 were considered statistically significant.

### Analytical specificity of LAMP primers

Specificity of the LAMP primers were tested using genomic DNAs (gDNAs) of various *Plasmodium *species (*P. knowlesi*, *Plasmodium falciparum, Plasmodium simium, Plasmodium cynomolgi, Plasmodium fragile, Plasmodium brasilianum*, *Plasmodium vivax*) by gel electrophoresis. The gDNAs were obtained from American Type Culture Collection (ATCC).

### Clinical sensitivity and specificity

The clinical sensitivity and specificity of LAMP assay was calculated using 74 whole-blood samples and microscopy as the reference standard method. Sensitivity was calculated as (number of true positives)/(number of true positives + number of false negatives), and specificity was calculated as (number of true negatives)/(number of true negatives+ number of false positives).

## Results

A total of 74 samples were included in this study to determine the sensitivity and specificity of LAMP method in detecting *P. knowlesi*.

### Specificity of LAMP primers

**Figure 1:**
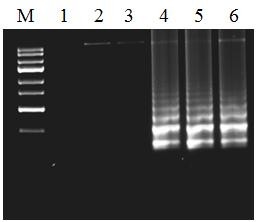
**Agarose gel electrophoresis of LAMP products**. Electrophoresis of loop-mediated isothermal amplification (LAMP) products amplified from genomic DNA and blood samples. Lane 1-3, negative controls of genomic DNA of *P. falciparum*, genomic DNA *P. vivax *and blood sample of healthy donor, respectively; lane 4, genomic DNA of *P. knowlesi*; lane 5-6, *P. knowlesi *positive blood samples and lane M, 100-bp DNA ladder.

Various plasmodium genomic DNAs comprising of 39 non-*knowlesi *malaria blood samples (*P. vivax*, n = 28; *P. falciparum*, n = 10; *P. malariae*, n = 1, as determined by BinaxNOW Malaria Kit and nested PCR) and 20 blood samples from healthy donors were used as template in this LAMP experiment to investigate the specificity of LAMP primers. All *P. knowlesi *positive samples produced a typical ladder of multiple bands on the agarose gel while other plasmodium and healthy donor samples did not produce such bands (Figure 1).

### Sensitivity of LAMP detection

LAMP is highly sensitive with the detection limit as low as ten copies for AMA-1 plasmid compared to nested PCR (100 copies). Clinical sensitivity of LAMP was compared with the results from conventional nested PCR and microscopy. By microscopy, a total of 13 *P. knowlesi *suspected samples (12 with either *P. malarie *or *P. knowlesi *and 1 with either *P. falciparum *or *P. knowlesi*) were detected positive for *P. knowlesi *under LAMP. However, nested PCR with species specific primers only detected 12 samples as positive for *P. knowlesi *while another sample showed no relation to other Plasmodium species (Table 2).

**Table 2:** Results of LAMP compared to microscopy and nested PCR for detecting *P. knowlesi*

Microscopy/nested PCR result	LAMP result		Microscopy/nested PCR total
		
	*P. knowlesi*	Negative	
*Microscopy result*			
*P. knowlesi *or *P. malariae *	12	1^a^	13
*P. knowlesi *or *P. falciparum*	1^c^	1^b^	2
Non-*knowlesi*	0	39	39
Negative	0	20	20
			
*Nested PCR result*			
*P. knowlesi*	12	0	12
Non-*knowlesi*	0	41	41
Negative	1^c^	20	21
			
LAMP total	13	61	74

^a ^Sample positive for *P. malariae *by nested PCR

^b ^Sample positive for *P. falciparum *by nested PCR

^c ^Sample positive for either *P. knowlesi *or *P. falciparum *by microscopy, with parasitaemia less than 0.01%

### Optimization of LAMP assay

Temperature of 65°C was found to be optimal in producing the best ladder pattern for positive *P. knowlesi *samples. Similar results were obtained when either a water bath or a heating block was used.

The LAMP assay was easy to conduct, although prevention of contamination proved necessary. Precautions such as changing of gloves between every PCR assay and different work areas for different parts of the experiment were taken into account.

## Discussion

Loop-mediated isothermal amplification (LAMP), the novel method developed by Notomi *et al *[4], is able to amplify DNA with great efficacy as its detection limit for DNA in the reaction mixture is as low as six copies, within an hour under isothermal conditions (65°C). LAMP is also an easy, convenient and cost-saving method, which only requires simple laboratory apparatus such as water bath or heating blocks to perform the test. Therefore, it has been widely applied as a diagnostic tool for several viral, bacterial, and parasitic diseases [7-9]. Han *et al *had reported a species-specific LAMP method for the diagnosis of four well-known human malaria *Plasmodium *species [10]. This study is the first report whereby LAMP assay is used for the diagnosis of the fifth human malaria *P. knowlesi *infection in human blood samples. In this study, *P. knowlesi *DNA was successfully amplified within an hour at 65°C by using LAMP primers that target the AMA gene of *P. knowlesi*.

Previous studies have indicated that LAMP assay has a higher sensitivity compared to nested PCR in detection of parasites, such as *Trypanosoma *spp. [11], *Babesia *spp. [12], and *T. gondii *[9]. The capability of LAMP assay to detect a single microfilaria in samples, as demonstrated by Aonuma *et al*, clearly shows that the method is highly sensitive [13]. In order to evaluate the sensitivity of LAMP assay for the detection of *P. knowlesi *in human samples, the LAMP assay was compared with conventional nested PCR, which targeted on *P. knowlesi *SSU gene and microscopic examination. Under microscopy, the early trophozoites of *P. knowlesi *morphologically resembled those of *P. falciparum*, while the late and mature trophozoites, schizonts and gametocytes of *P. knowlesi *in human infections were generally indistinguishable from those of *P. malariae*. Hence, the lack of distinguishable morphological features of *P. knowlesi *makes it extremely difficult to identify *P. knowlesi *infections by microscopy alone and laboratory misdiagnosis of *P. knowlesi *as *P. malariae *is inevitable [3].

In this study, 13 patient samples which were suspected to be either *P. knowlesi*/*P. malariae *or *P. knowlesi*/*P. falciparum *infections and were unable to be determined solely by microscopic examination, showed positive for *P. knowlesi *via LAMP assay, while *P. knowlesi *nested PCR was unable to detect one of these cases (12 positive as *P. knowlesi *infections among the 13 samples). The parasitaemia level of that particular case was found to be very low (< 0.01%). On the other hand, there were two cases whereby microscopic examination showed it to be *P. knowlesi*/*P. malariae *or *P. knowlesi/P. falciparum *infection but both LAMP and nested PCR showed negative for *P. knowlesi*. This might be due to misidentification by using microscopic examination as it requires training, skills and experience. These results showed that LAMP method which employs four primers and targets six distinct sequences on the *P. knowlesi *DNA could be used as a molecular confirmatory test of *P. knowlesi *infection instead of microscopic examination which is unable to differentiate the species among *P. knowlesi, P. malariae *and *P. falciparum*. Furthermore, it is more sensitive than nested PCR and the detection limit of LAMP assays is also lower relative to nested PCR.

Besides sensitivity, the high specificity of LAMP assay was demonstrated by screening genomic DNAs other *Plasmodium *species (*P. falciparum, P. simium, P. cynomolgi, Plasmodium fragile, P. brasilianum, Plasmodium inui and Plasmodium simiovale*), and extracted DNA from blood samples of other *Plasmodium *malaria and healthy donors. Results showed that LAMP assay which was specifically designed for *P. knowlesi *did not amplify DNAs of other *Plasmodium *spp. and no cross-reactivity occurred with all the negative controls. The result indicates that by using LAMP assays, *P. knowlesi *can be efficiently distinguished from other malaria parasites.

The simplicity and high efficiency of LAMP to amplify DNA under isothermal conditions within an hour suggests that LAMP could be a potential alternative for detection of *P. knowlesi *in samples compared to nested PCR, which requires an expensive PCR machine and is time-consuming. Another advantage of using LAMP assay is due to its turbidity-based detection of the positive reaction. The positive and negative amplifications could be distinguished by observing the turbidity of reactions with the naked eye, without using gel electrophoresis and therefore the result can be analysed within a few minutes [14]. Another way to analyse the LAMP result is by adding a DNA intercalating dye, SYBR green I to the end-products and visualizing it under UV light. The turbidity can be better observed under UV light. Besides that, the amplicon sequence was confirmed by sequence analysis to eliminate false positive and contaminated reactions.

There are however, limitations to this method. For visualization of results under UV light, the product tubes need to be opened for the dye to be added and this might lead to cross-contamination. This limitation can be overcome by practicing sterile pipetting techniques or using LAMP master mix, which contains pre-added fluorescence dye. Another drawback is the size of target DNA. The efficiency of LAMP is affected by the size of target DNA as the strand displacement DNA synthesis step limits the rate or amplification. The size of target DNA has to be lesser than 300 base pair in order to obtain satisfying results [4]. Hence, target DNA, which is larger than 500 bp is not suitable to be used as it will lead to poor amplification.

## Conclusion

Based on the results obtained, LAMP assays could be a potential alternative for molecular diagnosis and routine screening of *P. knowlesi *infection especially in malaria endemic countries, including Malaysia. It could also be useful in monitoring malaria control and eradication programmes.

## Competing interests

The authors declare that they have no competing interests.

## Authors' contributions

YLL, RM, PYC, VP, FWC, LCC and YC carried out laboratory work and analysed the data. LCC and AMA collected the blood samples. MYF and CNA participated in the data analyses and helped to draft the manuscript. All authors read and approved the final manuscript.
